# Supplementary material for: Multi‐dataset identification of innovative feature genes and molecular mechanisms in keratoconus
Source: J Cell Mol Med. 2024 Sep 19;28(18):e70079. doi: 10.1111/jcmm.70079 (PMC11412914; doi:10.1111/jcmm.70079)
Supplement: Supplementary file 2 — Material S2. [file JCMM-28-e70079-s002.zip › 7.GSEA analysis/6.SNORA74B/KEGG_FC_EPSILON_RI_SIGNALING_PATHWAY.html]

Details for gene set KEGG\_FC\_EPSILON\_RI\_SIGNALING\_PATHWAY[GSEA]

|  || Dataset | SNORA74B.group.cls#h\_versus\_l |
| Phenotype | group.cls#h\_versus\_l |
| Upregulated in class | l |
| GeneSet | KEGG\_FC\_EPSILON\_RI\_SIGNALING\_PATHWAY |
| Enrichment Score (ES) | -0.4406163 |
| Normalized Enrichment Score (NES) | -1.5473344 |
| Nominal p-value | 0.007889546 |
| FDR q-value | 0.3473028 |
| FWER p-Value | 0.699 |
Table: GSEA Results Summary

  

Fig 1: Enrichment plot: KEGG\_FC\_EPSILON\_RI\_SIGNALING\_PATHWAY      
 Profile of the Running ES Score & Positions of GeneSet Members on the Rank Ordered List

  

| SYMBOL | TITLE | RANK IN GENE LIST | RANK METRIC SCORE | RUNNING ES | CORE ENRICHMENT || 1 | PIK3R5 | na | 170 | 0.800 | 0.0594 | No |
| 2 | RAC3 | na | 1795 | 0.390 | 0.0439 | No |
| 3 | IL3 | na | 5142 | 0.221 | -0.0349 | No |
| 4 | PLA2G4E | na | 5383 | 0.212 | -0.0248 | No |
| 5 | SOS1 | na | 5971 | 0.193 | -0.0262 | No |
| 6 | INPP5D | na | 6064 | 0.190 | -0.0136 | No |
| 7 | PIK3R1 | na | 8116 | 0.133 | -0.0621 | No |
| 8 | PLCG2 | na | 8239 | 0.131 | -0.0551 | No |
| 9 | MAPK11 | na | 8296 | 0.129 | -0.0463 | No |
| 10 | IL13 | na | 9160 | 0.110 | -0.0624 | No |
| 11 | AKT2 | na | 9380 | 0.105 | -0.0603 | No |
| 12 | PLCG1 | na | 9672 | 0.099 | -0.0607 | No |
| 13 | KRAS | na | 9766 | 0.097 | -0.0555 | No |
| 14 | VAV2 | na | 10064 | 0.091 | -0.0568 | No |
| 15 | PLA2G12B | na | 10125 | 0.090 | -0.0513 | No |
| 16 | MAPK9 | na | 10427 | 0.085 | -0.0532 | No |
| 17 | PLA2G10 | na | 10767 | 0.079 | -0.0566 | No |
| 18 | MAPK13 | na | 11114 | 0.073 | -0.0607 | No |
| 19 | PDPK1 | na | 11353 | 0.069 | -0.0620 | No |
| 20 | MAPK12 | na | 11855 | 0.062 | -0.0715 | No |
| 21 | MAPK14 | na | 12827 | 0.049 | -0.0956 | No |
| 22 | PIK3R2 | na | 12946 | 0.047 | -0.0952 | No |
| 23 | PIK3CA | na | 13079 | 0.046 | -0.0953 | No |
| 24 | SOS2 | na | 13685 | 0.039 | -0.1096 | No |
| 25 | JMJD7-PLA2G4B | na | 13953 | 0.036 | -0.1144 | No |
| 26 | MAP2K2 | na | 14904 | 0.025 | -0.1397 | No |
| 27 | MAPK10 | na | 14996 | 0.024 | -0.1404 | No |
| 28 | PIK3CB | na | 15769 | 0.017 | -0.1613 | No |
| 29 | VAV3 | na | 16097 | 0.014 | -0.1696 | No |
| 30 | MAP2K4 | na | 16268 | 0.012 | -0.1736 | No |
| 31 | PLA2G12A | na | 16297 | 0.012 | -0.1735 | No |
| 32 | PLA2G6 | na | 16344 | 0.011 | -0.1739 | No |
| 33 | MAPK3 | na | 16753 | 0.007 | -0.1851 | No |
| 34 | PLA2G2F | na | 17001 | 0.005 | -0.1919 | No |
| 35 | MAP2K7 | na | 17007 | 0.005 | -0.1916 | No |
| 36 | PRKCD | na | 17017 | 0.005 | -0.1915 | No |
| 37 | AKT1 | na | 18366 | -0.010 | -0.2296 | No |
| 38 | BTK | na | 18390 | -0.011 | -0.2294 | No |
| 39 | PLA2G2E | na | 18608 | -0.013 | -0.2346 | No |
| 40 | PIK3CG | na | 18884 | -0.016 | -0.2413 | No |
| 41 | RAC2 | na | 18892 | -0.016 | -0.2402 | No |
| 42 | MAP2K3 | na | 18965 | -0.017 | -0.2410 | No |
| 43 | PLA2G2D | na | 19469 | -0.023 | -0.2537 | No |
| 44 | HRAS | na | 19699 | -0.025 | -0.2582 | No |
| 45 | SYK | na | 19828 | -0.027 | -0.2598 | No |
| 46 | RAC1 | na | 19881 | -0.027 | -0.2591 | No |
| 47 | PIK3CD | na | 20354 | -0.032 | -0.2702 | No |
| 48 | MAPK8 | na | 20414 | -0.033 | -0.2692 | No |
| 49 | MAPK1 | na | 20606 | -0.035 | -0.2719 | No |
| 50 | LAT | na | 20912 | -0.039 | -0.2776 | No |
| 51 | RAF1 | na | 21074 | -0.041 | -0.2789 | No |
| 52 | FCER1G | na | 22025 | -0.052 | -0.3022 | No |
| 53 | PRKCA | na | 22239 | -0.055 | -0.3039 | No |
| 54 | IL5 | na | 23454 | -0.072 | -0.3332 | No |
| 55 | PLA2G4A | na | 24085 | -0.082 | -0.3448 | No |
| 56 | GRB2 | na | 24997 | -0.098 | -0.3631 | No |
| 57 | PLA2G2C | na | 26678 | -0.132 | -0.4010 | No |
| 58 | PLA2G3 | na | 27299 | -0.147 | -0.4071 | No |
| 59 | LCP2 | na | 27345 | -0.148 | -0.3965 | No |
| 60 | NRAS | na | 27969 | -0.164 | -0.4013 | No |
| 61 | LYN | na | 29275 | -0.203 | -0.4227 | Yes |
| 62 | PIK3R3 | na | 29453 | -0.208 | -0.4110 | Yes |
| 63 | PLA2G5 | na | 30479 | -0.245 | -0.4210 | Yes |
| 64 | MS4A2 | na | 31010 | -0.268 | -0.4147 | Yes |
| 65 | MAP2K1 | na | 31129 | -0.275 | -0.3960 | Yes |
| 66 | TNF | na | 31738 | -0.308 | -0.3887 | Yes |
| 67 | MAP2K6 | na | 31770 | -0.311 | -0.3647 | Yes |
| 68 | GAB2 | na | 32350 | -0.344 | -0.3537 | Yes |
| 69 | CSF2 | na | 32846 | -0.380 | -0.3374 | Yes |
| 70 | IL4 | na | 32876 | -0.383 | -0.3074 | Yes |
| 71 | VAV1 | na | 32928 | -0.388 | -0.2777 | Yes |
| 72 | FYN | na | 33540 | -0.454 | -0.2588 | Yes |
| 73 | PRKCB | na | 33882 | -0.508 | -0.2278 | Yes |
| 74 | PLA2G2A | na | 33973 | -0.527 | -0.1880 | Yes |
| 75 | FCER1A | na | 33985 | -0.529 | -0.1458 | Yes |
| 76 | PLA2G1B | na | 34211 | -0.585 | -0.1053 | Yes |
| 77 | PRKCE | na | 34306 | -0.615 | -0.0586 | Yes |
| 78 | AKT3 | na | 34657 | -0.880 | 0.0020 | Yes |
Table: GSEA details [plain text format]

  

Fig 2: KEGG\_FC\_EPSILON\_RI\_SIGNALING\_PATHWAY      
 Blue-Pink O' Gram in the Space of the Analyzed GeneSet

  

Fig 3: KEGG\_FC\_EPSILON\_RI\_SIGNALING\_PATHWAY: Random ES distribution      
 Gene set null distribution of ES for **KEGG\_FC\_EPSILON\_RI\_SIGNALING\_PATHWAY**
